# Experiences of Overseas Trained Physical Therapists Working in Saudi Arabia: An Observational Study

**DOI:** 10.3390/ijerph17103406

**Published:** 2020-05-13

**Authors:** Ahmad H. Alghadir, Hamayun Zafar, Zaheen A. Iqbal

**Affiliations:** 1 Rehabilitation Research Chair, College of Applied Medical Sciences, King Saud University, Riyadh 11433, Saudi Arabia; aha@ksu.edu.sa (A.H.A.); hz@ksu.edu.sa (H.Z.); 2 Department of Odontology, Umea University, 90187 Umea, Sweden

**Keywords:** experiences, physical therapists, Saudi Arabia, migration, health care

## Abstract

*Background:* Health professionals, including physical therapists (PTs), are known to migrate for better jobs, as well as for personal and professional development. However, this involves risks of maladjustment, discrimination, and exploitation. We conducted this study to investigate the experiences of overseas trained PTs in Saudi Arabia (SA) and their perceptions about physical therapy practice and problems regarding the profession in the country. *Methods*: A questionnaire and accompanying explanation of the study was sent to 175 members of the Saudi Physical Therapy Association (SPTA) working in SA who had been trained outside SA and had a minimum of one year of work experience before moving to SA. *Results*: One hundred and fifty (86%) respondents completed the questionnaire. Among the respondents, the majority had more than five years of work experience after moving to SA. While 54% of the respondents reported that they were satisfied with their work experiences in SA, the remaining respondents reported their dissatisfaction for various reasons. *Conclusions*: With the increase in aged population and rise in disability, the need for PTs has simultaneously increased in the health care sector around the world, including in SA. Until PTs of local origin are ready to fill the requirements, the services of PTs from other countries will be required in SA. Although the majority of respondents reported having positive work experiences in SA, the negative aspects and challenges faced by PTs in SA have also been highlighted in this study. These problems need to be addressed in order to promote the development of a better and more holistic approach to patient care.

## 1. Introduction

Health care professionals, including physical therapists (PTs), nurses, etc., have been known to migrate to different countries, not only in search of better jobs and for their personal, social, and professional development, but also to fill the work force requirement [1]. Along with the potential benefits, this migration also involves various risks, such as difficulty in adjustment, discrimination, exploitation, etc. [2,3]. During 2009, the allied health care population increased by 43% in the UK, which included 3000 PTs [4].

Physical therapy education is influenced by diverse health care policies and cultures around the world, and different countries follow different curriculums and guidelines during the training of PTs [5]. Such non-uniformity makes it difficult for PTs to adapt in a new environment after migration [6,7]. Various studies have reported on the experiences of health care professionals, especially nurses and doctors, working outside their home country [3,7,8]. Some studies have reported positive experiences [5,9], while most of the studies have focused on the difficulties faced by health care professionals working abroad [10,11]. Although PTs comprise a major part of the migrated health care population around the world, studies about their experiences and the problems they face are fewer, and to the best of our knowledge, there have been no previous studies conducted in the Middle East region.

Saudi Arabia (SA) is one of the fastest growing economies in the world. According to the 2013 government census, 33% of the population of SA consists of an expatriate workforce working in different fields, including health care. Physical therapy has been identified as the most important branch of the health care profession in SA [12,13], and PTs constitute the maximum number of staff working in the rehabilitation sector, out of which around 80% are employed by the government in hospitals [14]. We conducted an observational study using a self-administered survey questionnaire to find answers to the following questions. What are the experiences of overseas trained PTs working in SA? What are their perceptions about physical therapy practice and the problems of the profession in the country? The objective of this study was to identify areas to improving patient care and the approach of the PTs towards their patients in SA.

## 2. Materials and Methods

### 2.1. Design

A 30-item structured questionnaire was designed based on similar studies [5,6,7,15] to explore the experiences of overseas trained PTs working in SA. It included three domains: demographic and professional characteristics; the experiences of PTs working outside their home country; and the perception of physical therapy education, profession, and training in SA. Also, respondents were asked to rate their work satisfaction on a ten-point scale.

The questionnaire was first presented to a group of five local senior PTs for a pilot study. After receiving the results, minor changes to the structure and language were executed so that it would be well received by the respondents and so that its outcome would fulfill the aims and objectives of the study. The study was designed so that it would take respondents only 3–5 minutes to answer. The language of the questionnaire was English, and its summary is described in Table 1. 

### 2.2. Participants

Physical therapy professionals who were members of the Saudi Physical Therapy Association (SPTA) and were involved in direct patient contact for more than 20 hours per week were eligible to participate in the survey. They had to be trained as a physical therapist outside SA and must have had a minimum of one year of work experience before moving to SA. The participants had to complete the questionnaire online. The questionnaire was uploaded online and its link, along with an explanation of the purpose of the study, was sent to the 175 members of the SPTA working in SA. Respondents were assured of the confidentiality of their information and were requested to complete the questionnaire within one month. Two weeks after uploading the questionnaire online, a reminder email was sent. Incomplete questionnaires were rejected.

### 2.3. Statistical Analysis

The Statistical Package for Social sciences program (SPSS) version 21 (IBM Inc., Armonk, NY, USA) was used for data analysis. Descriptive statistics, including frequencies and percentages, were used for demographic data, level of satisfaction, and perceptions of overseas trained PTs.

### 2.4. Ethics Approval and Consent to Participate

All participants were informed about the purpose and nature of this study, and their written informed consent was obtained. Ethical approval in compliance with the Helsinki Declaration as revised in 2013 was obtained from the Rehabilitation Research Review Board, King Saud University.

### 2.5. Availability of Data and Material

The datasets used in this study are available from the corresponding author on reasonable request.

## 3. Results

Out of the 175 SPTA members that were sent the questionnaire, 150 (86%) respondents completed the questionnaire. However, only 125 (83%) of the responses were eligible according to the inclusion and exclusion criteria of the study. The respondents represented 11 different nationalities who reported having been trained in different parts of the world (Table 2).

### 3.1. Demographic and Professional Characteristics

The mean age of the respondents was 34.66 (±6.63) years. Among the 125 respondents, 84 (67%) had a bachelor’s degree in physical therapy, while 17 (14%) and 4 (2%) also had a master’s degree and a PhD, respectively. The majority of respondents (*n* = 32; 26%) reported that they were working in a general specialty, with 51 (41%) respondents working as senior physical therapists in various clinics and hospitals. Among the 125 respondents, 51 (41%) of the respondents had more than five years of work experience before moving to SA, while 42 (34%) reported more than five years of work experience after moving to SA. Table 2 shows the detailed demographic and professional characteristics of the respondents.

### 3.2. Working outside Their Home Country

The majority of the respondents (*n* = 39; 31%) reported that they decided to migrate for work outside their home country for their professional development, and 35 (28%) reported that they chose SA due to jobs that offered a better salary compared to their home country. Out of the 125 respondents, 68 (54%) of the respondents were satisfied with working in SA, as their experience matched their expectations. Among these, 23 (34%) respondents reported experiencing a friendly and supportive work environment, 20 (29%) appreciated the opportunity to experience a different culture, and 15 (22%) liked the availability of educational and career opportunities (Table 3).

Respondents who reported that they were not satisfied with their work in SA gave various reasons to support themselves. These reasons included poor remuneration (*n* = 20; 35%), cultural shock (*n* = 10; 18%), and communication barriers with patients and colleagues (*n* = 7; 12%), among other reasons mentioned in Table 3. Experiences of racism and bullying were also reported by 51 (41%) respondents. Out of 125 respondents, 44 (35%) also reported a lack of equal opportunities and 19 (15%) reported discrimination with regard to career progression as compared to PTs of local origin. One hundred and three respondents (82%) reported that their working hours were too long and 21 (17%) reported that they were unable to handle their clinical load. Around half of the respondents claimed that they had to change their area of work after moving to SA, and 88 (70%) respondents reported that they never received any incentives or promotions after starting work in SA. On a scale of 10, 10 (8%) respondents marked their level of satisfaction as less than 3, 61 (49%) marked their level of satisfaction as between 3 and 5, while 54 (43 %) marked their level of satisfaction as more than 5 (Table 3).

### 3.3. Perception of Physical Therapy Education, Profession, and Training in Saudi Arabia

In response to a question regarding the status of physical therapy education in SA, 17 (14%) indicated that it was superior in comparison to its level in the country where they were trained, while the remaining respondents reported that it was either the same or inferior. Forty-seven (38%) respondents reported that the duration of the physical therapy degree in SA is the same as their country of education. Regarding the level of prestige of physical therapy as a profession in SA, 22 (18%) reported that it was higher compared to their home country, while the remaining 103 (82%) reported that it was either lower or the same (Table 4).

Around 16 (13%) respondents found the physical therapy approach to patients in SA to be more holistic (Table 4). Eighty-four (67%) respondents found their local colleagues to be supportive and helpful. Seventy (56%) respondents reported that they were happy with their professional status and autonomy in SA, and 66 (53%) reported that they frequently got the opportunity to update their knowledge through conferences and seminars. Seventy-four (59%) respondents complained about the difficulties they faced to register with the health council to obtain work license. Despite the majority of respondents reporting to be happy with their work life in SA, 99 (79%) of the respondents said they have plans to return their home country in the future.

## 4. Discussion

Physical therapy was introduced to SA universities a little later than the rest of the world [16]. Due to a lack of PTs of local origin, foreign staffs were hired at universities and hospitals in SA to meet the high patient demand. Although there are many studies in the literature that report on the experiences of nurses and doctors working outside their country, studies about the experiences and problems faced by PTs are fewer, and to the best of our knowledge, there have been no previous studies conducted in the Middle East region. We conducted an observational study to investigate the experiences of overseas trained PTs working in SA by using a self-administered online questionnaire. Fifty-four percent of the respondents reported that they were satisfied with their work life in SA, while others had a reason or two for reporting otherwise.

Around 7% of the respondents were Saudi in origin but had been educated or trained outside their country. It has been the practice of the Saudi Arabian government to fund students who want to get training outside their country, especially at the master’s and PhD level [16]. Expatriates need extensive knowledge and skills to adjust to a new work environment in another country [5]. Although physical therapy education varies around the world, more than 50% of respondents reported that they found that the level of physical therapy education in SA was the same as their country. Professional support and a helpful work culture have been linked to successful adaptation to a new work environment [3,17]. Having your opinion counted in your team and among your seniors empowers self-identities and helps with gaining confidence, respect, and responsibility [5]. Besides these factors, frequent chances of updating their skills and knowledge through various conferences and seminars as well as supportive and helpful colleagues of local origin are the other reasons reported by the respondents behind their adaptation and reports of being happy with their professional status and autonomy. SPTA regularly organizes workshops, seminars, and conferences with a prime focus on professional development and improvement of all its members practicing in the country, thereby enabling them to update their knowledge and improve the care of their patients [16].

However, not being in first contact with the patients, getting written prescriptions from physicians, and an inability to advise investigations for patients were reasons the respondents reportedfor being unhappy. Similar reasons have also been reported by PTs working in the UK [6,15], where loss of professional freedom has been referred to as a loss of professional autonomy that limits the extent to which they are able to practice independently. Only 13% of respondents reported that they found the approach to patients in SA more holistic than other countries. The introduction of a self-referral scheme, as introduced by the Department of Health in the UK [5], may help solve this problem.

Around 51% of the respondents reported that they had at least one experience of racial discrimination or bullying after moving to SA. Other types of discriminations reported included lack of equal opportunities and career progression (reported by 35% and 15% of respondents, respectively). Similar experiences have been reported by nurses, dentists, and doctors working around the world outside of their home countries [1,18,19]. Other reasons that respondents listed for their work failing to meet their original expectations were a high work load and poor financial remuneration. Such experiences have been reported to affect patient care [10]; as such, the government should take care of such incidents in order to allow for better outcomes.

SA is a country with a closed culture where males and females are highly segregated. Most of the respondents came from different cultures where there are no restrictions with regard to genders, and where therapists are free to treat male or female patients and patients are free to choose their therapists. Approximately 18% of the respondents gave this reason for not adjusting to the work environment in SA and reported experiencing a cultural shock when they first landed in SA. The Saudi Commission for Health Specialties conducts licensure examinations for local and expatriate physical therapists in order to qualify them to practice [16]. The licensing process includes a theoretical examination as well as practical examination in order to ensure a high quality of patient care [20]. Around 59% of the respondents reported that they considered this step to be a difficult and long process. The prevalence of work-related low back pain was reported to be high among physical therapists working in Riyadh, which could be another reason for dissatisfaction among them [21]. Prevention strategies including the introduction of ergonomics, coping strategies to reduce stress, and the promotion of teamwork during their training is recommended.

This is the first study of its kind in the Middle East. Many of the references cited in this study are older, as the literature search returned no recent related results. This also indicates the novelty of the study. A self-reported questionnaire that has not been widely used in the literature was used in this study. This could have encouraged respondents to overestimate their responses and thus the responses may not be reflective of the respondents’ true thoughts.

It is recommended that a similar study using a validated questionnaire aimed at determining job satisfaction should be conducted in various different countries simultaneously. This would also help to determine which factors are the most important to PTs when choosing a country for employment. It should also address the verification of the responses coming from the respondents.

## 5. Conclusions

With an increasingly aging population and rise in disability, the need for PTs has simultaneously increased in health care sectors around the world, including SA. Until PTs of local origin are ready to fill the requirement, the services of professionals from other countries will be required in SA. Although the majority of respondents had reported having positive work experiences in SA, negative aspects and challenges faced by PTs in SA have also been highlighted in this study. These problems need to be addressed in order to promote the development of a better and more holistic approach to patient care.

## Figures and Tables

**Table 1:** A questionnaire consisting of 30 items was divided into three domains.

Domains of Questionnaire	Components
Demographic and professional characteristics	Nationality Age Last degree obtained and specialization Country from where physical therapy degree was obtained Work experience in home country and Saudi Arabia
Experiences of PTs working outside their home country	Level of work satisfaction Reasons for choosing to work in Saudi Arabia Patient load Any racism or discrimination Fulfilled expectations
The perception of physical therapy education, profession, and training in Saudi Arabia	Status of physical therapy as a profession in Saudi Arabia compared to the country the physical therapy was trained in Physical therapy education system in Saudi Arabia Licensing process for registration in Saudi Arabia

PTs: physical therapist.

**Table 2:** Demographic and professional characteristics of respondents. Percentages are provided in parentheses.

**Nationality**					
Philippines	Jordan	Saudi Arabia	Egypt	India	Others
51 (41)	16 (13)	9 (7)	12 (10)	14 (11)	23 (18)
**Last qualification**					
Diploma	Bachelor’s degree		Master’s degree		PhD
21 (17)	84 (67)		17 (14)		3 (2)
**Country of study/training**					
Egypt	India	Jordan	Philippines	Syria	Others
13 (10)	15 (12)	15 (12)	52 (42)	5 (4)	25 (20)
**Specialization**					
General	Orthopedics	Neurology	Cardio- pulmonary	Sports	Others
32 (26)	28 (22)	24 (19)	7 (6)	8 (6)	26 (21)
**Work experience before moving Saudi Arabia**					
<1 year	1–5 years		>5 years		
00 (00)	74 (59)		51 (41)		
**Work experience after moving Saudi Arabia**					
<1 year	1–5 years		>5 years		
15 (12)	66 (53)		42 (34)		
**Current work designation**					
PT assistant	Junior PT	Senior PT	Consultant		Other
20 (16)	27 (22)	51 (41)	7 (6)		20 (16)

PT: physical therapist.

**Table 3:** Level of satisfaction among respondents regarding working outside their home country. Percentages are provided in parentheses.

**Reason for choosing Saudi Arabia to work**	
Back to home country	16 (13)
Personal and social growth	25 (20)
Professional development	39 (31)
Better salary compared to home country	35 (28)
Travel opportunity	4 (3)
Other	4 (3)
**After moving to Saudi Arabia, did your experience match your expectations?**	
Yes	68 (54)
No	57 (46)
**If yes, why?**	
Friendly and supportive work environment	23 (34)
Availability of educational and career opportunities	15 (22)
Appreciation of the opportunity to experience a different culture	20 (29)
Others	10 (15)
**If no, why?**	
Communication barrier with patients and colleagues	7 (12)
High workload	3 (5)
Lack of professional development	12 (21)
Poor financial remuneration	20 (35)
Cultural shock	10 (18)
Others	5 (9)
**Did you have any experience of racism or bullying?**	
Yes	51 (41)
No	74 (59)
**Any discrimination with regard to…?**	
Lack of equal opportunities	44 (35)
Career progression	19 (15)
Training	9 (7)
None	53 (42)
**Are your working hours…?**	
Too long?	103 (82)
Too short?	22 (18)
**Are you able to handle your patient load in your clinic?**	
Yes	104 (83)
No	21 (17)
**Did you have to change your area of work after moving to Saudi Arabia?**	
Yes	62 (50)
No	63 (50)
**Do you get frequent incentives and promotion?**	
Yes	37 (30)
No	88 (70)
**On a scale of 10, what is your current level of satisfaction?**	
<3	10 (8%)
3–5	61 (49%)
>5	54 (43%)

**Table 4:** Experience of respondents regarding physical therapy education, profession, and training in Saudi Arabia. Percentages are provided in parentheses.

**How do you find the level of prestige of physical therapy as a profession in Saudi Arabia compared to the country you were trained in?**	
Higher	22 (18)
Lower	49 (39)
Same	54 (43)
**How do you find the status of physical therapy education in Saudi Arabia compared to the country where you were trained?**	
Inferior	45 (36)
Superior	17 (14)
Same	63 (50)
**Does the physical therapy education system in Saudi Arabia have the same duration as your country of education?**	
Yes	47 (38)
Duration of degree is more	24 (19)
Duration of degree is less	54 (43)
**How do you find approach of physical therapists to patient in Saudi Arabia?**	
Less holistic	13 (10)
More holistic	16 (13)
Multi-disciplinary	35 (28)
Independent	32 (26)
None	29 (23)
**Are your local colleagues supportive and helpful?**	
Yes	84 (67)
No	41 (33)
**Are you happy with your professional status and autonomy?**	
Yes	70 (56)
No	55 (44)
**How did you find the licensing process in Saudi Arabia?**	
Difficult	74 (59)
Easy	51 (41)
**Do you get opportunities for continuing your education in Saudi Arabia?**	
Yes	66 (53)
No	59 (47)
**Do you have any plan to return to your home country?**	
Yes	99 (79)
No	26 (21)

## Abbreviations

PTs	physical therapists
SA	Saudi Arabia
SPTA	Saudi Physical Therapy Association

## References

[1] Kingma M. (2008). Nurses on the move: Diversity and the work environment. Contemp. Nurse.

[2] Kingma M. (2008). Nurse Migration and the Global Health Care Economy. Policy Politics Nurs. Pr..

[3] Nichols J., Campbell J. (2010). The experiences of internationally recruited nurses in the UK (1995–2007): An integrative review. J. Clin. Nurs..

[4] Young R., Noble J., Mahon A., Maxted M., Grant J., Sibbald B. (2010). Evaluation of international recruitment of health professionals in England. J. Health Serv. Res. Policy.

[5] Kyle H., Kuisma R. (2013). The experiences of overseas trained physiotherapists working in the United Kingdom National Health Service. Physiotherapy.

[6] Moran A., Nancarrow S., Butler A. (2005). There’s no place like home a pilot study of perspectives of international health and social care professionals working in the UK. Aust. N. Z. Health Policy.

[7] Foo J., Storr M., Maloney S. (2016). Registration factors that limit international mobility of people holding physiotherapy qualifications: A systematic review. Health Policy.

[8] Virdee S. (2014). Racism, Class and the Racialized Outsider.

[9] Alexis O., Vydelingum V., Robbins I. (2007). Engaging with a new reality: Experiences of overseas minority ethnic nurses in the NHS. J. Clin. Nurs..

[10] Allan T.H. (2010). Mentoring overseas nurses: Barriers to effective and non-discriminatory mentoring practices. Nurs. Ethic.

[11] Hunt B. (2007). Managing equality and cultural diversity in the health workforce. J. Clin. Nurs..

[12] Alshami A.M., Al Maghraby M.A. (2013). Learning style and teaching method preferences of Saudi students of physical therapy. J. Fam. Community Med..

[13] Bindawas S.M. (2014). Physical Therapy Entry-level Education and Post-professional Training in Saudi Arabia: A Comparison of Perceptions of Physical Therapists from Five Regions. J. Phys. Ther. Sci..

[14] Alghahtani A., Barhameen A., AbuAbat A. (2007). Saudi Strategic Plan for Rehabilitation Services.

[15] Turner P. (2001). The occupational prestige of physiotherapy: Perceptions of student physiotherapists in Australia. Aust. J. Physiother..

[16] Alghadir A., Zafar H., Iqbal Z.A., Anwer S. (2015). Physical therapy education in Saudi Arabia. J. Phys. Ther. Sci..

[17] Alexis O. (2009). Overseas trained nurses’ perception of UK nurses’ caring attitudes: A qualitative study. Int. J. Nurs. Pr..

[18] Larsen J. (2007). Embodiment of discrimination and overseas nurses’ career progression. J. Clin. Nurs..

[19] Alghadir A., Zafar H., Iqbal Z.A. (2015). Work-related musculoskeletal disorders among dental professionals in Saudi Arabia. J. Phys. Ther. Sci..

[20] (2009). Professional Classification Manual for Health Practitioners.

[21] Alghadir A., Zafar H., Iqbal Z.A., Al-Eisa E. (2017). Work-Related Low Back Pain Among Physical Therapists in Riyadh, Saudi Arabia. Work. Health Saf..

